# Direct evidence for the semipersistent transmission of *Cucurbit chlorotic yellows virus* by a whitefly vector

**DOI:** 10.1038/srep36604

**Published:** 2016-11-04

**Authors:** Jingjing Li, Xiangzhi Liang, Xueli Wang, Yan Shi, Qinsheng Gu, Yen-Wen Kuo, Bryce W. Falk, Fengming Yan

**Affiliations:** 1 College of Plant Protection, Henan Agricultural University, Zhengzhou, Henan 450002, China; 2 Zhengzhou Fruit Research Institute, Chinese Academy of Agricultural Sciences, Zhengzhou, Henan 450009, China; 3 Department of Plant Pathology, University of California, Davis, One shields Avenue, Davis, CA 95616-8600, USA

## Abstract

*Cucurbit chlorotic yellows virus* (CCYV) (genus *Crinivirus*, family *Closteroviridae*) is an emerging plant virus, and is now spreading and causing severe economic losses to cucurbit crops in many Asian countries. CCYV is believed to be transmitted specifically by the sweetpotato whitefly, *Bemisia tabaci*, in a semipersistent manner. In the present study, we provide direct evidence for the semipersistent transmission of CCYV by Mediterranean (MED) cryptic species of *B. tabaci* complex. We investigated CCYV transmission characteristics, and immunofluorescently labeled and localized the virus retention site within the vector by laser confocal microscopy. Whiteflies required ≥1 h of acquisition access period (AAP) to successfully acquire CCYV, and the proportion of RT-PCR positive whitefly individuals reached to 100% at 48 h of AAP. CCYV virons could be retained within vectors as long as 12 d, but the proportion of RT-PCR positive whiteflies dropped to 55% by 3 d. Groups of thirty whiteflies given a 24 h of inoculation access period (IAP) to inoculate CCYV on cucumber plants showed a transmission efficiency rate of 72.73%. The retention site of CCYV virons was located in the foregut of virion-fed vectors. These results definitely indicated the semipersistent transmission mode of CCYV by *B. tabaci* MED.

Due to a strong cell wall boundary and immobility of plants, most plant viruses need vectors for the transmission to new host plants or to a new habitat^1^. Hemipterans, because of their piercing-sucking mouthparts and special feeding behaviors, are the most common vectors for transmission of plant viruses^2^,^3^. Insect vectors transmit plant viruses in different ways depending on the combination of characteristics of the viruses, vectors and plants. These transmission types are generally categorized into nonpersistent, semipersistent and persistent modes, according to the length of the period the vector can harbor infectious particles, which can range from minutes to hours (nonpersistent), to days (semipersistent) and to life-time and even inheritance by the insect progeny (persistent-propagative)^4^,^5^,^6^.

Virus retention sites within vectors vary depending on type of virus, vector and transmission modes. Viruses that are transmitted in a nonpersistent manner are retained in the stylet^4^,^7^, while viruses transmitted in a persistent manner are circulative-non-propagative or circulative-propagative, i.e., they move with plant sap into the vector gut, from the gut lumen into the hemolymph or other tissues and finally back into the salivary glands from which they are introduced back into the plant during insect feeding^6^, or even can pass directly through the sheath of the filter chamber and be readily transmitted to healthy plants, as the case of *Wheat dwarf virus* (WDV) in its leafhopper vector, *Psammotettix alienus*^8^. Compared to the viruses having nonpersistent or persistent transmission relationships, transmission characteristics and retention sites of viruses transmitted in a semipersistent manner are less understood^7^. It is generally believed that viruses transmitted in a semipersistent manner are retained in the foregut of the vector, as experimentally shown for *Lettuce infectious yellows virus* (LIYV, genus *Crinivirus*) with unique membrane feeding and immunofluorescent localization^9^,^10^. But Hogenhout *et al*.^6^ pointed out that biological transmission characteristics are not always correlated with retention site of virus within the insect vector, so the retention sites within vectors and strategies still need to be identified for some nonpersistent or semipersistent viruses, especially for the newly reported viruses^7^.

*Cucurbit chlorotic yellows virus* (CCYV) is a newly identified virus species of the genus *Crinivirus* within the family *Closteroviridae*^11^, and is transmitted specifically by cryptic species Middle East-Asia Minor 1 (MEAM1, previously known as B biotype) and Mediterranean (MED, previously known as Q biotype) of the sweetpotato or tobacco whitefly, *Bemisia tabaci* (Gennadius) (Hemiptera: Aleyrodidae)^12^. CCYV was firstly identified in melon plants in Japan, and is now widespread throughout China, Sudan, Lebanon, Iran and Egypt^12^,^13^,^14^,^15^,^16^,^17^,^18^,^19^. CCYV can cause chlorotic leaf spots and complete yellowing of leaves in cucumber, melon, watermelon, pumpkin, loofah plants and *Nicotiana benthamiana*, resulting in yield and economic losses^12^. Moreover, it was reported that CCYV and CYSDV (*Cucurbit yellow stunting disorder virus*) showed mixed infections in Lebanon^20^. Studies on CCYV were mainly on the detection methods and plant resistance breeding^12^,^21^,^22^, but the details of the transmission process and the virus retention site within its vectors are still unclear^20^.

In this study, we investigated CCYV transmission characteristics by *B. tabaci* MED cryptic species, i.e., transmission efficiency, acquisition access period (AAP), retention time within the vector, and inoculation access period (IAP). Meanwhile, we used a membrane feeding and immunofluorescent labeling^9^ to localize the specific retention of virions within the whitefly vector. These results provide direct evidence for the semipersistent transmission of CCYV and establish the basis for further research on vector-virus interactions and for implementation of effective strategies for control of the virus and insect vectors.

## Results

### Detection of CCYV in individual whiteflies

**Figure 1:**
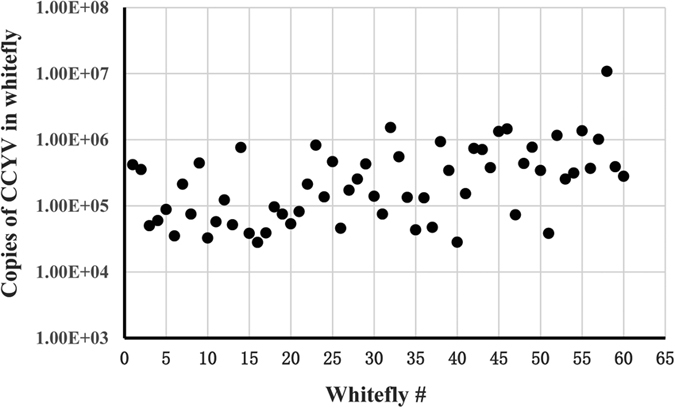
Copies of CCYV RNA in individual whiteflies of *Bemisia tabaci* MED. Whiteflies were allowed to feed on CCYV-infected cucumber plants for 3 d, and 60 whiteflies were individually subjected to real- time RT-PCR test. Data (0 copies) for non-viruliferous whiteflies (control) were not shown. In the *Y*-axis, 1.00E + 05 = 10^5^.

We used real-time RT-PCR to detect CCYV RNA copy numbers of individual whiteflies having fed on virus-infected cucumber plants for 3 d. The results showed that 100% whitefly individuals after 3 d feeding on CCYV-infected plants had acquired CCYV with amounts ranging from 1.34 × 10^4^ to 1.09 × 10^7^ copies with 95% of individual whiteflies carrying 10^4^–10^6^ copies (Fig. 1). No CCYV RNA was detected in control group of individual whiteflies feeding on non-infected cucumber plants (data not shown in Fig. 1).

### Transmission efficiency

**Figure 2:**
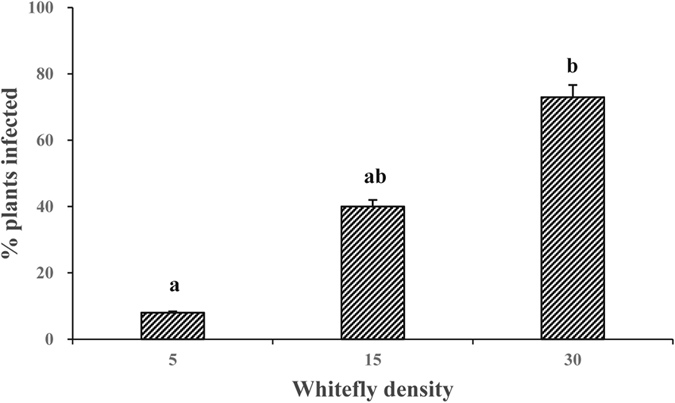
The effects of *B. tabaci* MED number on the proportion of CCYV-infected plants. After feeding on CCYV-infected cucumber plants for 3 days, groups of 5, 15 and 30 whiteflies were respectively placed on non-infected cucumber leaves (3–4 leaf stage) with clip cages for 24 h. Twenty four plants were used for each whitefly group with 3 replicates, and plants were analyzed by RT-PCR after 30 days. Data shown are mean ± SE.

After feeding on CCYV-infected cucumber plants for 3 days, groups of 5, 15 and 30 whiteflies of *B. tabaci* MED cryptic species were respectively placed on non-infected cucumber leaves (3–4 leaf stage) with clip cages for 24 h. After 30 days, plant symptoms were checked and the infection status was analyzed with RT-PCR. The results showed that the efficiency of CCYV transmission was positively related to the number of whiteflies used for inoculation. After 24 h of inoculation on healthy cucumber plants, groups of 30 whiteflies transmitted CCYV with the efficiency of 72.73%, while groups of 15 and 5 whiteflies only transmitted CCYV to 40% and 8% plants, respectively (Fig. 2).

### Acquisition access period

**Figure 3:**
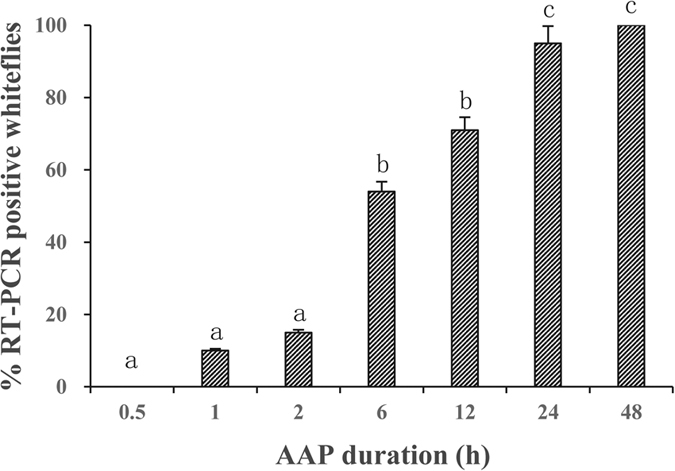
The effects of acquisition access period (AAP) on the proportion of RT-PCR positive adults of *B. tabaci* MED. Newly emerged whiteflies were allowed to feed on CCYV-infected cucumber plants for 0.5, 1, 2, 6, 12, 24 and 48 h, respectively, and 24 whiteflies from each group were collected for real-time RT-PCR tests. Three replicates were used. Data shown are mean ± SE.

Newly emerged whitefly adults of *B. tabaci* MED cryptic species were allowed to feed on CCYV-infected cucumber plants for 0.5, 1, 2, 6, 12, 24 and 48 h, respectively, and 24 whiteflies from each group were collected for real-time RT-PCR tests. The proportion of RT-PCR positive whiteflies increased with the time of acquisition access period (AAP) when whiteflies fed on CCYV-infected cucumber plants, so AAP significantly affected the percentage of RT-PCR positive whiteflies (*F* = 33.66, *df* = 6, 147, *P* < 0.0001). Whiteflies required ≥1 h of AAP to acquire CCYV virons successfully, and the proportion of RT-PCR positive whiteflies reached to ca. 55% at 6 h AAP, and to 100% at 48 h of AAP (Fig. 3).

### Retention time

**Figure 4:**
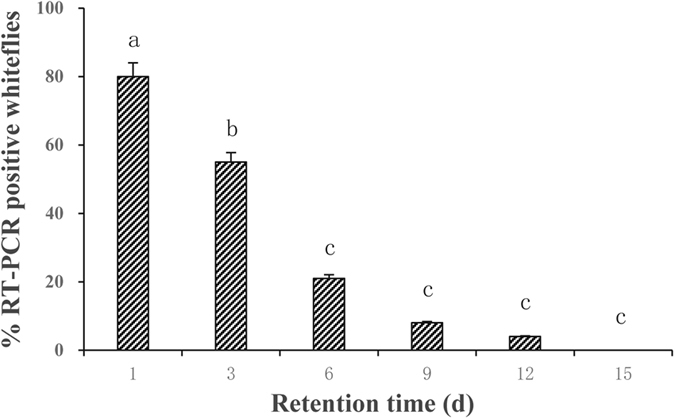
The effects of retention time on the proportion of RT-PCR positive *B. tabaci* MED. Whiteflies having fed on CCYV-infected cucumber plants for 3 days were transferred to cotton plants (non-host plants for CCYV but host plant for whiteflies). Twenty-four whiteflies were collected at time intervals of 1, 3, 6, 9, 12 and 15 d, respectively, for real-time RT-PCR tests. Data shown are mean ± SE. Three replicates were used for each collection.

After 3 d feeding on CCYV-infected plants, adults of *B. tabaci* MED were transferred to cotton plants (cotton is the host plant for whiteflies, but non-host plant for CCYV) for determination of the retention time. Whiteflies collected at time intervals of 1, 3, 6, 9, 12 and 15 d, respectively, were subject for real-time RT-PCR tests. The results showed that the proportions of RT-PCR positive whiteflies decreased with time (*F* = 19.36, df = 5, 130, *P* < 0.0001) (Fig. 4). CCYV could be retained in *B. tabaci* as long as 12 d, but the proportion of RT-PCR positive whiteflies dropped to 55% on 3rd d and even lower level on 6th d or later.

### Inoculation access period

**Figure 5:**
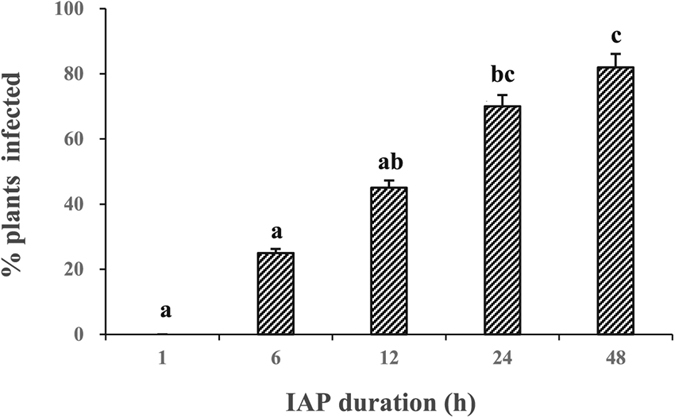
The effects of inoculation access period (IAP) of the vector *B. tabaci* MED on the proportion of CCYV-infected cucumber plants. After feeding CCYV-infected cucumber plants with 3 days, 30 whiteflies were placed on non-infected cucumber leaves (3–4 leaf stage) with clip cages for 1, 6, 12, 24 and 48 h, respectively. Twenty-four plants were used for each treatment and detected with RT-PCR after 30 days. Data shown are mean ± SE. Three replicates were used.

Thirty whiteflies, having fed on CCYV-infected cucumber plants for 3 days, were placed on non-infected cucumber leaves (3–4 leaf stage) with clip cages for inoculation access period (IAP) of 1, 6, 12, 24 and 48 h, respectively. After 30 days, 24 plants used for each inoculation time treatment were detected with RT-PCR. We found that CCYV transmission efficiency of *B. tabaci* was positively correlated with IAP (*F* = 7.50, df = 4, 51, *P* < 0.0001) (Fig. 5). Groups of 30 whiteflies were unable to transmit CCYV within 1 h IAP, but CCYV was transmissible after 6 h inoculation, though the transmission efficiency was still low. The transmission efficiency for groups of 30 whiteflies after 24 h and 48 h of IAP was 70.00% and 81.82%, respectively.

### Localization of CCYV virions within whitefly vectors

**Figure 6:**
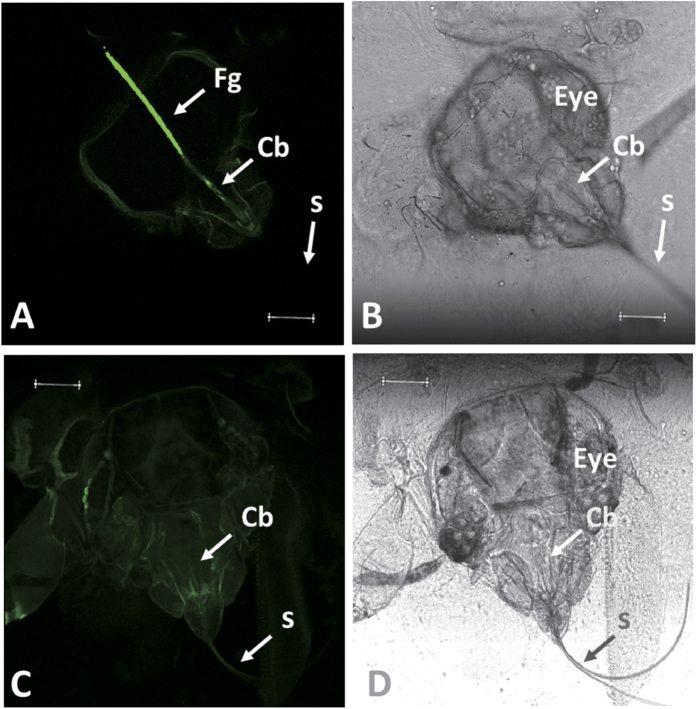
Retention site localization of CCYV virions in the foregut and cibarium of *B. tabaci* MED under confocal laser scanning microscopy (Leica, TCS SP8). (**A**) Confocal view of the head of viruliferous *B. tabaci* adult, with background transimitted light blocked. (**B**) Transmitted light view of *A*. (**C**) Confocal view of the head of non-viruliferous *B. tabaci* adult, with background transimitted light blocked. (**D**) Transmitted light view of *C*. Under confocal laser scanning microscopy, we observed non-viruliferous (having fed on healthy cucumber plants) or viruliferous whiteflies (having fed on CCYV-infected cucumber plants) after sequential membrane feeding of the following solutions: (i) basal diet, (ii) diet containing anti-CCYV-CP IgG, and (iii) diet containing a goat anti-rabbit IgG conjugated with Alexa Fluor 488. The presence of fluorescent signals showing the retention site within the vector was indicated with the arrow (Fg). The eye, foregut (Fg), cibarium (Cb), and stylets (S) are indicated. (Scale bars, 50 μm).

To determine the retention site of CCYV virions within its whitefly vector, we used a membrane feeding approach to give viruliferous whiteflies sequential access to basal artificial liquid diet containing anti-CCYV-CP IgG and goat anti-rabbit IgG conjugated with Alexa Fluor 488^9^. Then we observed the dissected heads or foreguts of whiteflies under confocal laser scanning microscopy (Leica, TCS SP8). The results revealed the presence of bright green fluorescent signals distributed in the cibarium, anterior pharynx and posterior esophagus (generically referred to as the foregut) within vectors, showing that the retention site of CCYV virions within the whitefly is the foregut (Fig. 6).

## Discussion

As a member of the genus *Crinivirus*, CCYV is believed to be transmitted by whitefly vectors in a semipersistent mode^12^, but the transmission details have so far been lacking. In this study, we provided direct evidence for the semipersistent transmission of CCYV by investigating transmission characteristics and localizing the virus retention site within the vector.

Transmission efficiency is variable with virus species and their vectors. Criniviruses occur at low titer and are restricted to the phloem of infected plants^9^, therefore whiteflies take some time to reach the phloem to acquire viruses and inoculate viruses to new host plants, and the transmission efficiency is vector-number dependent. Whitefly numbers less than 30 generally are not efficient for *Crinivirus* transmission. In our study, the efficiency of CCYV transmission by 30 *B. tabaci* MED whiteflies after a 24 h of IAP was 72.73% (Fig. 2). Similarly, 40 whiteflies *B. tabaci* A biotype had efficiency of 74.30% for LCV (*Lettuce chlorosis virus*) transmission^23^. But transmission of CYSDV (*Cucurbit yellow stunting disorder virus*) by 30 adults of *B. tabaci* A biotype, SqVYV (*Squash vein yellowing virus*) by 30 adults of *B. tabaci* B biotype (MEAM1), and LCV by 40 adults of *B. tabaci* B biotype had only efficiency of 47.00%^24^, 57.00%^25^ and 57.5%^23^, respectively.

In our study, *B. tabaci* MED whiteflies required a minimum 1 h AAP to acquire detectable CCYV, while 1 h AAP for LCV^23^ and 2 h for CYSDV^24^, but only 10 min for LIYV^23^. In our experiments, whiteflies within 1 h IAP were unable to transmit CCYV. The minimum IAP for inoculation of CCYV to new host plants by 30 whitefly was 6 h, much longer than those for SqVYV (30 min)^25^, LIYV (*Lettuce infectious yellows virus*) (1 h), LCV (1 h), and CYSDV (2 h). For these viruses above, 48 h IAP was enough for the vector whiteflies to obtain 80% or more transmission efficiency.

It was reported that criniviruses like CYSDV, LIYV and LCV can be harbored in the vectors for ca. 10 days^26^. Our results showed that *B. tabaci* MED can harbor CCYV as long as 12 d when analyzed using real-time RT-PCR. The vectors of CYSDV, LIYV and LCV retained the ability to transmit the respective virus to plants for 3 or 4 days, and it can be presumed that *B. tabaci* MED whiteflies can not transmit CCYV after 3 or 5 days because the percentage of RT-PCR positive whiteflies was ca. 20% on 6^th^ day.

Semipersistently transmitted viruses can be retained in the stylet or foregut of insect vectors^9^,^10^,^27^. Viruses of the genus *Crinivirus*, by virtue of their semipersistent transmission characteristics, have been postulated to be retained in the foreguts, and clearly the work of Chen *et al*.^9^ showed this to be the case for LIYV. By contrast, *Cauliflower mosaic virus* (CaMV) was shown to be retained at the stylet tip of their aphid vectors^28^, but CaMV differs from most viruses that are transmitted in a semipersistent manner in that it readily moves through mesophyll tissues of infected plants while criniviruses are limited to the phloem, and CaMV can be acquired from and inoculated to plants by probing, vs. feeding as the means for criniviruses. While the retention sites for many criniviruses remains to be identified^7^, here we found that CCYV is retained in the foregut within the whitefly vector, *B. tabaci* MED cryptic species, by using the membrane feeding and immunofluorescent localization approach (Fig. 6). Our results are very similar to those of Chen *et al*.^9^ for LIYV.

In conclusion, our results have shown that CCYV transmission characteristics and retention within the vector are consistent with a semipersistent type of transmission. Future work includes comparison of transmission capacity between two cryptic species (MED and MEAM1) of *B. tabaci* and molecular mechanisms. In the previous experiment we found that MED is more effective in transmission of CCYV than MEAM1 (unpublished data), and studies of protein-protein interactions between virus and vector may reveal the mechanisms of difference in transmission ability^9^,^29^.

## Materials and Methods

### Maintenance of plants, whiteflies, and virus clones

The colony of *B. tabaci* Mediterranean (MED) cryptic species (formerly referred to as Q biotype) was maintained on tobacco plants (*Nicotiana tabacum* cv. Zhongyan-100) in cages (60 cm × 40 cm × 80 cm) in a greenhouse at 28 ± 0.5 °C, 16:8 h LD and 75 ± 0.5% relative humidity. The genetic purity of *B. tabaci* MED cultures was monitored every 3–5 generations using the random amplified polymorphic DNA polymerase chain reaction (RAPD-PCR) technique combined with the sequencing of *mtCO1* gene^30^. Plants of cucumber (*Cucumis sativus* cv. Youlu-10) were used for virus clone maintenance and transmission assays. Cotton (*Gossypium hirsutum* L. cv. Yinshan-1) is the suitable host plants of *B. tabaci* but not of CCYV, so it was used for the retention time experiment in transmission assays (see below). Viruliferous *B. tabaci* MED adults were collected from CCYV-infected melon plants and transferred to cucumber plants, and cucumber plants were inoculated with CCYV by viruliferous *B. tabaci* MED adults and maintained in growth chambers under above-mentioned conditions.

### Detection of CCYV virions in the whitefly vectors

#### RNA extraction and synthesis of cDNA

Total RNA of individual adult whiteflies from infected cucumber plants was extracted using TRIzol reagent (Invitrogen) following the manufacturer’s instructions. RNA concentration and purity were measured in a NanoDrop^TM^ spectrophotomer (Thermo Scientific) and stored at −20 °C for subsequent analysis. Total RNA (1 μg) from each sample was reverse transcribed to generate the first-strand cDNA using the PrimeScript^TM^ RT reagent Kit (Takara).

#### Primer design

Primers were designed based on CCYV coat protein coding sequences by using primer premier 5 software and the nucleotide sequence in GenBank (accession no. HM581658.1). The forward primer (CCYV-F: 5′-GCGACCATCATCTACAGGCA-3′; nucleotide positions 548–567) and the reverse primer (CCYV-R: 5′-CCGACTTGTTCCTTTCAGAGC-3′; nucleotide positions 679–699) were found to have an optimal annealing temperature of 60 °C. The primer pair generates a 152 base pair fragment. Subsequent Primer-BLAST searches showed that they had a high specificity towards CCYV.

#### SYBR Green real-time RT-PCR

The real-time RT-PCR assays were performed using SYBR Premix Ex Taq^TM^ II (Takara). All reactions were carried out in a final volume of 20 μl each containing: 10 μl of SYBR Premix Ex Taq^TM^ II (Takara), 1 μl of cDNA or plasmid dilutions and 0.8 μl of each primer (CCYV-F and CCYV-R). Amplification reactions were performed as follows: 94 °C for 2 min; 40 cycles of 94 °C for 15 s, 60 °C for 20 s, 72 °C for 20 s.

For generation of standard quantification curves, serial 10-fold dilutions of plasmid (3.79 × 10^2^ to 3.79 × 10^8^ copies/μl) were used as templates for real-time RT-PCR. Standard curves were obtained by linear regression analysis of the threshold cycle (Ct) value of each of three replicates over the logarithm of the copy numbers present in each sample. To determine the sensitivity and the reliability of the real-time RT-PCR, three individual assays were undertaken using the 10-fold serial dilutions of plasmid.

### Assays for virus transmission

#### Transmission efficiency

Thousands of whiteflies were placed into the insect-proof cage containing two CCYV-infected cucumber plants. Three days after feeding, groups of 5, 15 and 30 whiteflies were transferred in clip cages^31^ onto the underside of healthy leaves of cucumber plants (3–4 leaf stage), respectively. Twenty-four plants were used for each whitefly density treatment with 3 replicates. After 24-h IAP, clip cages were removed and whiteflies were collected for CCYV detection, while the plants were treated with insecticide (imidacloprid) and maintained in whitefly- free greenhouse to test with RT-PCR after 30 days.

#### Acquisition access period (AAP)

Ca. 500 non-viruliferous whiteflies were placed on two CCYV-infected cucumber plants in the insect-proof cage. Twelve whiteflies (12 per plant) were collected at time intervals following introduction of CCYV-infected cucumber plants: 0.5, 1, 2, 6, 12, 24, 48 h. Whiteflies feeding on non-infected cucumbers served as negative controls. Three replicates were used. The total RNA of individual whiteflies was extracted using Trizol reagent, and detected by real-time RT-PCR.

#### Retention time

Hundreds of whiteflies were released into the insect-proof cage containing two CCYV-infected cucumber plants for feeding. Three days after feeding, about 500 whiteflies were collected and starved for 2–3 h before putting into another cage containing two cotton plants (CCYV non-host plants) for feeding. Whiteflies (12 per plant) were collected at time intervals of 1, 3, 6, 9, 12 and 15 d after feeding. Whiteflies feeding on healthy cotton plants for 3 days were served as negative controls. The total RNA of individual whiteflies was extracted using Trizol reagent, and analyzed by real-time RT-PCR. Twenty-four whiteflies were tested for each collection with 3 replicates.

#### Inoculation access period (IAP)

Thousands of whiteflies were put into the insect-proof cage containing two CCYV-infected cucumber plants for feeding. Three days after feeding, 30 whiteflies which were starved for 2–3 hours, were placed in clip cages clipped in the back of non-infected cucumber leaves (3–4 leaf stage). Clip cages were removed at time intervals of 1, 6, 12, 24 and 48 hours after feeding, and whiteflies were collected for CCYV detection, while the plants were treated with insecticide (imidacloprid) and maintained in the whitefly-free greenhouse. Twenty-four plants were used for each treatment with 3 replicates. Whiteflies feeding on non-infected cucumber plants served as negative controls.

### Location of virion retention site within the vector

Two groups of *B. tabaci* MED adults were used: one group fed on CCYV-infected cucumber plants whereas the other group fed on healthy cucumber plants. Adult whiteflies were placed in membrane feeding cages, with approximately 50–100 whiteflies per cage. Whiteflies were first fed with basal artificial liquid diet [15% sucrose and 1% BSA in 1× TE (10 mM Tris-HCl, 1 mM EDTA, pH 7.4)] to remove unbound or nonspecifically bound virions, and then fed with basal artificial liquid diet containing anti-CCYV-CP polyclonal IgG raised in rabbit (1/500-fold dilution of 0.74 mg/mL), followed by basal artificial liquid diet containing a 1/200-fold dilution of goat anti-rabbit IgG conjugated with Alexa Fluor 488 (Invitrogen). Whiteflies were allowed to feed on each of the above solutions for 10- to 12-h. To remove unbound or nonspecifically bound antibodies present in the ingested solutions, whiteflies were fed with a basal artificial diet for several hours after feeding of antibody-containing solutions^9^. We observed the dissected heads or foreguts of whiteflies under confocal laser scanning microscopy (Leica, TCS SP8).

### Statistical analyses

In experiments of AAP and retention time, numbers of RT-PCR positive whiteflies and copies of CCYV were recorded and calculated, and proportions of RT-PCR positive whiteflies were calculated by numbers of RT-PCR positive over test whiteflies which did not include the failure ones for RNA extraction. In experiments of efficiency of transmission and IAP, numbers of infected plants were recorded and proportions of infected plants were calculated by numbers of infected plants over test plants which did not include the dead ones in the process of growth. Based on these data, variance analysis with Duncan’s new multiple range method was carried out using IBM Statistical SPSS software 20.0.

## Additional Information

**How to cite this article**: Li, J. *et al*. Direct evidence for the semipersistent transmission of *Cucurbit chlorotic yellows* *virus* by a whitefly vector. *Sci. Rep*. **6**, 36604; doi: 10.1038/srep36604 (2016).

**Publisher’s note:** Springer Nature remains neutral with regard to jurisdictional claims in published maps and institutional affiliations.
